# Esophageal cooling for protection during left atrial ablation: a systematic review and meta-analysis

**DOI:** 10.1007/s10840-019-00661-5

**Published:** 2019-11-22

**Authors:** Lisa WM Leung, Mark M Gallagher, Pasquale Santangeli, Cory Tschabrunn, Jose M Guerra, Bieito Campos, Jamal Hayat, Folefac Atem, Steven Mickelsen, Erik Kulstad

**Affiliations:** 1 Department of Cardiology, St George’s University Hospitals NHS Foundation Trust, St. George’s, University of London, Cranmer Terrace, Tooting, London, SW17 0RE UK; 2 Perelman School of Medicine, University of Pennsylvania, 3400 Civic Center Blvd, PA 19104 Philadelphia, United States; 3 Hospital de la Santa Creu I Sant Pau, Universitat Autònoma de Barcelona, CIBERCV, Carrer de Sant Quintí, 89, 08041 Barcelona, Spain; 4 Department of Gastroenterology, St George’s University Hospitals NHS Foundation Trust, St. George’s, University of London, Cranmer Terrace, Tooting, London, SW17 0RE UK; 5 Department of Neurology and Neurotherapeutics, UT Southwestern Medical Center, 5323 Harry Hines Blvd, 75390 Dallas, TX United States; 6 Department of Internal Medicine, University of Iowa Carver College of Medicine, 200 Hawkins Drive, 52242 Iowa City, United States; 7 Department of Emergency Medicine, UT Southwestern Medical Center, 5323 Harry Hines Blvd, 75390 Dallas, TX United States

**Keywords:** Atrial fibrillation, Radiofrequency ablation, Esophageal injury, Esophageal cooling, Atrio-esophageal fistula

## Abstract

**Purpose:**

Thermal damage to the esophagus is a risk from radiofrequency (RF) ablation of the left atrium for the treatment of atrial fibrillation (AF). The most extreme type of thermal injury results in atrio-esophageal fistula (AEF) and a correspondingly high mortality rate. Various strategies for reducing esophageal injury have been developed, including power reduction, esophageal deviation, and esophageal cooling. One method of esophageal cooling involves the direct instillation of cold water or saline into the esophagus during RF ablation. Although this method provides limited heat-extraction capacity, studies of it have suggested potential benefit. We sought to perform a meta-analysis of published studies evaluating the use of esophageal cooling via direct liquid instillation for the reduction of thermal injury during RF ablation.

**Methods:**

We searched PubMed for studies that used esophageal cooling to protect the esophagus from thermal injury during RF ablation. We then performed a meta-analysis using a random effects model to calculate estimated effect size with 95% confidence intervals, with an outcome of esophageal lesions stratified by severity, as determined by post-procedure endoscopy.

**Results:**

A total of 9 studies were identified and reviewed. After excluding preclinical and mathematical model studies, 3 were included in the meta-analysis, totaling 494 patients. Esophageal cooling showed a tendency to shift lesion severity downward, such that total lesions did not show a statistically significant change (OR 0.6, 95% CI 0.15 to 2.38). For high-grade lesions, a significant OR of 0.39 (95% CI 0.17 to 0.89) in favor of esophageal cooling was found, suggesting that esophageal cooling, even with a low-capacity thermal extraction technique, reduces the severity of lesions resulting from RF ablation.

**Conclusions:**

Esophageal cooling reduces the severity of the lesions that may result from RF ablation, even when relatively low heat extraction methods are used, such as the direct instillation of small volumes of cold liquid. Further investigation of this approach is warranted, particularly with higher heat extraction capacity techniques.

## Introduction

Thermal damage to the esophagus is a risk from radiofrequency (RF) ablation or cryoablation of the left atrium for the treatment of atrial fibrillation (AF) [1–3]. The most extreme type of thermal injury is an atrio-esophageal fistula (AEF), with a mortality rate of 80% or more [4–8]. Various strategies for protecting the esophagus during RF ablation or reducing the severity of injury have been developed, including power reduction, avoidance of greater contact force, temperature monitoring, esophageal deviation, and esophageal cooling, with varying degrees of success [9–11].

Esophageal cooling for the purpose of protecting the esophagus during RF ablation has been investigated in multiple studies [12–20]. The techniques used have included the insertion of expandable balloon devices or cooling sacs that circulate water, and the direct instillation of ice-cold water or saline into the esophagus. The study designs have included animal models and mathematical models as well as human clinical studies. Most of the human clinical studies have used direct instillation of ice cold water or saline as the cooling method, and for this reason, we performed a meta-analysis of the data obtained in these studies to examine their range of effect sizes and estimate the potential efficacy of esophageal cooling for protection during RF ablation.

## Methods

### Data sources and search strategy

Using PubMed, we searched the literature dated from 1985 (prior to the earliest reports of endocardial ablation to treat atrial fibrillation) to June 2019 for studies published on esophageal cooling during cardiac ablation. We conducted a broad search with the following Boolean structure: (esophag* OR oesophag*) AND cooling AND (ablation OR fibrillation). We did not restrict the search to studies published in English only. Details of the systematic review were submitted for registration in PROSPERO on June 21, 2019, with further details describing the statistical plan added on September 11, 2019.

### Eligibility criteria

We excluded preclinical studies, bench-top, agar phantom, and mathematical model studies, and studies that did not include formal endoscopy as an outcome measure.

### Data collection

The primary data of interest were esophageal lesions identified endoscopically after RF ablation. Because we anticipated inconsistency in the categorization of lesion severity, we aimed to simplify all lesion severity measurement into severe lesions characterized by the presence of ulceration and mild to moderate lesions encompassing all other abnormalities. Studies identified were then assessed for quality using the Newcastle Ottawa Scale, which evaluates three quality parameters (selection, comparability, and outcome) divided across eight specific items. Each item on the scale is scored with up to one point, except for comparability, which can be adapted to the specific topic of interest to score up to two points, such that the maximum for each study is 9, with studies having less than 5 points being identified as representing a high risk of bias [21].

### Statistical analysis

For this meta-analysis, we input the study data into Review Manager 5.3, and we present the results graphically. SAS version 9.4 (SAS Institute Inc., Cary, NC, USA) was used for additional analyses. The Cochran–Mantel–Haenszel (CMH) method was employed to test the null hypothesis that the response rate is the same for the two arms (control versus treatment), after adjusting for possible differences in study response rates. Furthermore, we fitted a random effect model using SAS procedures GLIMMIX and NLMIXED by treating the studies as a random effect. Because lesion grades are often considered to be dichotomized into those that are likely to progress to AEF and those that are not, we initially analyzed the data as a binary outcome (high-grade lesions concerning for progression versus low grade lesions likely to heal spontaneously). Then, to further estimate effect size, we used an ordinal logistic random intercept model, taking into account the ordered nature of lesion grading (low to high, numerically).

## Results

We identified 9 studies using the above criteria. Five of these studies were excluded for being non-clinical. Berjano et al. utilized a finite element model in three dimensions to investigate the effects of a cooled intraesophageal balloon [12]. Lequerica et al. performed studies using an agar phantom-based model that was built to provide temperature readings at points between the esophageal lumen and the myocardium [13, 14]. Arruda et al. studied a custom developed system utilizing temperature-controlled saline or water in an in vitro lamb heart and esophagus preparation, followed by an in vivo model with six dogs [15]. Scanavacca et al. presented a study of the use of a saline filled esophageal balloon to attempt esophageal protection in a dog model [17]. A clinical study of 8 patients by Tsuchiya et al. was excluded for not using endoscopy to determine the presence of lesions after RF ablation [16].

The remaining 3 studies included a total of 494 patients. Details of these studies and the characteristics of the patients are shown in Tables 1, 2, 3.Because the manner of grading lesions varied among the studies, we incorporated the scales used in the three studies into a common stratification (Grades I, II, III, and IV).

**Table 1:** Characteristics of included studies, population demographics, and comorbidities

Study	Design	Number of patients	Patient age	Gender (% male)	AF subtype (% paroxysmal)	BMI	HTN	Diabetes	CHF	Ejection fraction
John et al.	Prospective observational	76	63.5 +/− 10	63.2%	40.8%	30.5 +/− 5.1	68.4%	18.7%	26.3%	55.5 +/− 9.1%
Kuwahara et al.	Randomized control	100	63 +/− 8.7	40.0%	32.0%	24 +/− 2.0	N/A	N/A	N/A	64 +/− 6.6%
Sohara et al.	Prospective observational	318	63.3 +/− 7.9	72.6%	57.2%	N/A	14.8%	4.1%	N/A	66.8 +/− 6.8%

**Table 2:** Ablation techniques and characteristics for each included study

Study	Ablation technology	Power	Mapping technology	Ablation type	Average Contact Force	Mean RF time per lesion (seconds)	Anesthesia	Endoscopy timing
John et al.	RF open-irrigation, 3.5-mm-tip, 8-Fr, force-sensing catheter (SmartTouch)	24–28 W	CARTO mapping system and Pentaray NAV multipolar mapping catheter	Bilateral antral PVI, creation of a left atrial roof and floor line, and ablation across the mitral isthmus	10 g	12.3 +/− 5.3	General	Within 24 h of the procedure
Kuwahara et al.	3.5 mm irrigated-tip ablation catheter (Thermocool, Biosense Webster, Inc.)	25–30 W	CARTO system	Circumferential PVI with focal ablation, and creation of left atrial roof or mitral isthmus lines with posterior wall isolation in non-paroxysmal AF	N/A	N/A	Conscious sedation	Within 24 h of the procedure
Sohara et al.	12F radiofrequency hot balloon catheter (Hayama Arrhythmia Institute, Kanagawa, Japan)	N/A	CARTO system	Balloon-based box isolation	N/A	N/A	General	Within 3 days of the procedure

**Table 3:** Characteristics of temperature monitoring and esophageal cooling utilized in each included study

Study	Temperature sensor type	Temperature sensor characteristics	Gastric tube type	Coolant	Cooling threshold	Follow-up duration
John et al.	18-Fr esophageal temperature probe (400 series M1024215, GE Healthcare, Chicago, IL)	Tube diameter 6 mm, cuff diameter 8.8 mm, single thermistor inside of the cuff at the distal tip	18-Fr orogastric tube (nasogastric sump tube 0046180, Bard, Inc., Covington, GA)	20 mL ice-cold saline	0.5 °C increase in temperature from baseline	N/A
Kuwahara et al.	A multi-thermocouple temperature probe (Sensitherm, St Jude Medical)	Three thermocouples; one of which was placed at the same level of the esophagus as the site of RF energy delivery on the LA posterior wall	Unspecified gastric tube	5 mL ice-water (0 °C)	42 °C temperature peak	Up to 8 weeks
Sohara et al.	Thermocouple thermometer (Delta Ohm, Caselle di Selvazzano, PD, Italy)	Thermal sensor of a deflectable, 4-mm tip ablation catheter	Unspecified gastric tube coated with xylocaine jelly	10–20 mL ionized contrast medium (Gastrografin) or nonionized low osmotic contrast medium (iopamidol) diluted 1:2 with physiologic saline refrigerated to about 10 °C	43 °C temperature peak (group B) or 39 °C temperature peak (group C)	>10 months minimum, mean 3.6 years

**Fig. 1:**
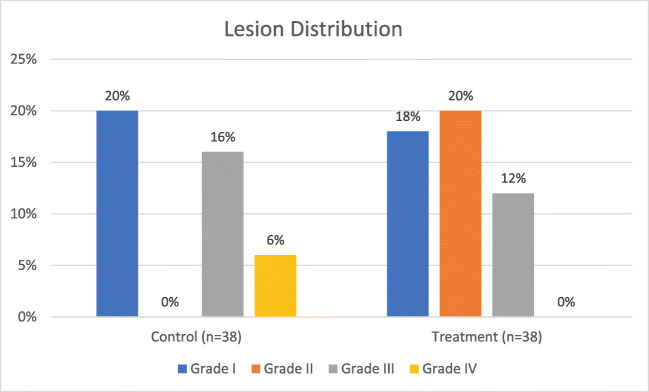
Results from John et al. Patients in the treatment group were actively cooled by injecting a 20 mL bolus of ice-cold saline via orogastric tube into the upper esophagus if/when the LET increased by 0.5 °C above baseline. Grade III and grade IV lesions are shown separately

John et al. studied 76 patients, half of which were actively cooled by injecting a 20 mL bolus of ice-cold saline via orogastric tube into the upper esophagus if/when the luminal esophageal temperature (LET) increased by 0.5 °C above baseline [20]. The authors found that this method of esophageal cooling did not decrease the overall incidence of thermal lesions, but noted a trend toward fewer severe lesions with cooling (Fig 1). The authors graded lesions as follows: grade 0, no esophageal lesion; grade 1, mucosal damage <1 cm width; grade 2, mucosal damage 1–3 cm width; grade 3, mucosal damage >3 cm width or visualization of deeper layer; and grade 4, bleeding ulcer or with overlying clot. Assessment of the study quality resulted in a score of 8 using the Newcastle Ottawa Scale.

**Fig. 2:**
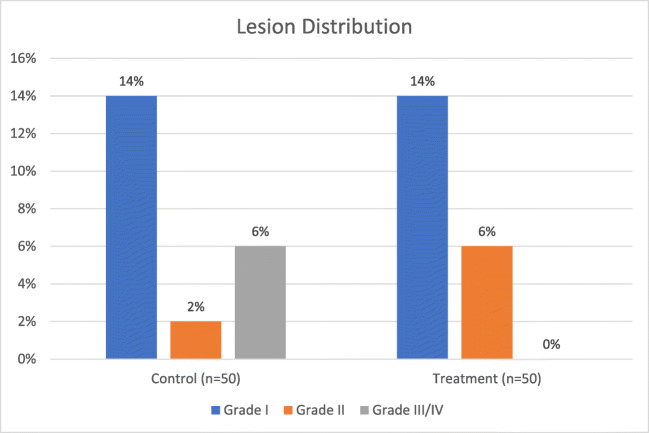
Results from Kuwahara et al. Patients in the treatment group were actively cooled by injecting 5 mL of ice water prior to RF energy delivery and subsequently when the LET reached 42 °C. The Grade III/IV lesion category represents all lesions qualitatively graded as “severe,” with mild lesions in Grade I and moderate lesions in Grade II

Kuwahara et al. studied 100 patients using very small volumes (5 mL) of ice water as the coolant for half of them. This volume was injected prior to RF energy delivery as well as subsequently if/when the LET reached 42 °C [18]. The authors found that this approach reduced the severity of esophageal lesions, but did not reduce the incidence: lesions occurred in 20% of the treatment group and 22% of the controls, with 3 moderate and 7 mild in the cooled group and 3 severe, 1 moderate, and 7 mild in the control group (Fig 2). The authors graded the severity of the lesions as mild, moderate, or severe, according to their extent and color. Assessment of the study quality resulted in a score of 8 using the Newcastle Ottawa Scale.

**Fig. 3:**
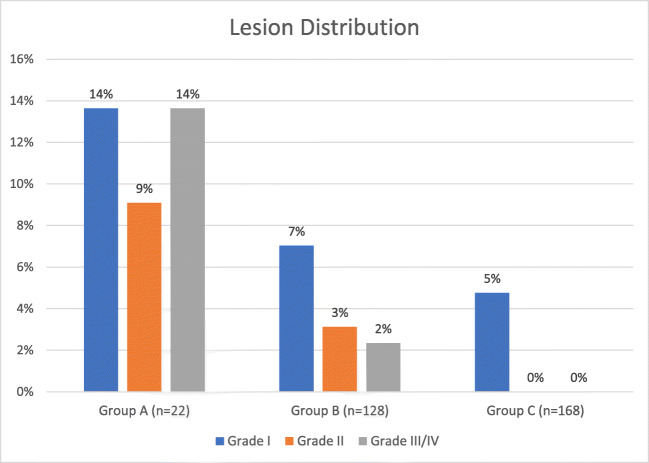
Results from Sohara et al. Patients in group A received only LET monitoring without cooling of the esophagus. Patients in groups B and C received LET monitoring with esophageal cooling when the LET exceeded 43 °C and 39 °C, respectively. Cooling was by infusion of cooled saline mixed with Gastrografin. The Grade III/IV lesion category represents all lesions graded as ulcers (scored as 3 or 4 by Sohara et al.)

Sohara et al. studied 318 consecutive patients divided into three groups, one receiving only temperature monitoring without cooling, the second receiving temperature monitoring with cooling when the LET exceeded 43 °C, and the third receiving temperature monitoring with cooling when the LET exceeded 39 °C. These authors used cooled saline mixed with Gastrografin or iopamidol as the coolant. The total volumes injected were slightly higher than those used by John et al. and Kuwahara et al. but were still limited (10–20 mL in repeated injected aliquots with a temperature of approximately 10 °C).[19] The percentage of patients free from any ulceration or erosion in each group was found to be 63.6%, 87.5%, and 95.2%, respectively (Fig 3). The authors classified the lesions as normal (score 1), erosion (patchy mucosal ulceration: score 2), mild ulcer (necrosis less than 3 mm in diameter with red spot: score 3), severe ulcer (necrosis more than 3 mm in diameter with red spot and/or with a hemorrhagic appearance, often with fibrinoid material: score 4). Assessment of the study quality resulted in a score of 9 using the Newcastle Ottawa Scale.

The studies by Kuwahara et al. and John et al. show a clear shift from high-grade to lower-grade lesions between the control and treatment arms [18, 20]. In contrast, the data from Sohara et al. show a general reduction in lesions of all grades [19].

**Fig. 4.**
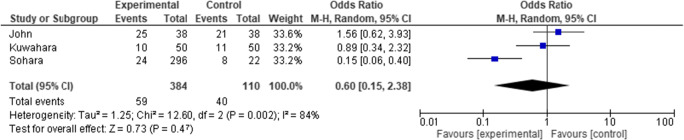
Forest plot comparing the outcome of all lesions in the three clinical studies. Events are the occurrence of grade I, II, III, and IV lesions

The forest plot comparing the outcome of all lesions (grades I, II, III, and IV) as events between control and treatment arms is shown in Fig 4. Although fewer lesions occurred in the treatment arms of the Sohara et al. and Kuwahara et al. studies, in meta-analysis this decrease did not reach statistical significance (OR 0.6, 95% CI 0.15 to 2.38).

**Fig. 5.**
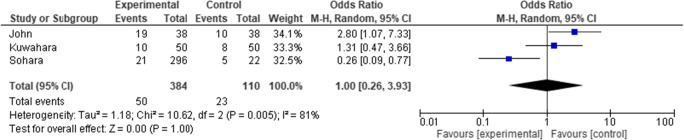
Forest plot comparing the outcome of low-grade lesions in the three clinical studies. Events are the occurrence of grade I and II lesions

The slight increase in low-grade lesions seen in the John et al. and Kuwahara et al. studies is shown in the forest plot in Fig 5, with an OR of 1.0 (95% CI 0.26 to 3.93). The number of low-grade lesions is not significantly impacted with this treatment.

**Fig. 6:**
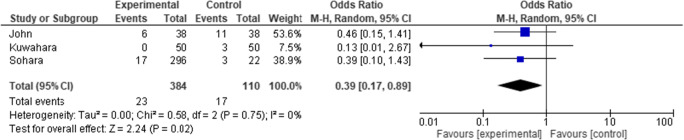
Forest plot comparing the outcome of severe lesions. Events are the occurrence of grade III/IV lesions

Evaluating the occurrence of high-grade lesions (grade III and IV) results in the forest plot shown in Fig 6, demonstrating a significant OR of 0.39 (95% CI 0.17 to 0.89) in favor of the treatment arm. Separately, using the CMH method, we obtained a significant *p* value of 0.016 indicating that the association between treatment and lesion grade remains strong. Furthermore, in a binary logistic regression model, an OR of 0.46 (95% CI 0.28 to 0.75) was found.

The I^2^ statistic, which describes the percentage of total variation across studies that is due to heterogeneity rather than chance, is shown in each of Figs. 4, 5, and 6. For the analysis using an outcome of severe lesions (Fig. 6), the I^2^ = 0%, which indicates no observed heterogeneity. Increasing heterogeneity of effect is seen (shown in the forest plots) when looking at the outcome of all lesions, and low-grade lesions, in Figs. 4 and 5, respectively.

Finally, using an ordinal logistic random intercept model rather than dichotomized outcome, we used GLIMMIX and NLMIXED in SAS to obtain an additional estimate of effect size for each category of lesion independently. This method showed that esophageal cooling by the method of direct instillation of cold water or saline used in these studies results in a point estimate of a −23% reduction in lesion grade, with 95% CI ranging from −85% to +38%.

## Discussion

Esophageal injury from RF ablation remains a feared complication in the treatment of atrial fibrillation, and a variety of techniques have been developed to reduce this risk. Esophageal cooling has shown promise in a number of studies, with such cooling being brought about by the instillation of cold water or saline directly into the esophagus via orogastric tube. Even with the relatively high heat capacity of water, the amount of thermal energy that can actually be absorbed by this method is limited by the low total volumes of liquid instilled. Nevertheless, our meta-analysis of three studies that used this approach suggests that cooling in this manner offers a clinically significant protective effect from severe lesions, and provides a 61% reduction in high-grade lesion formation (with a 95% CI of 11% to 83% reduction).

Prototypes of various balloon devices have been evaluated for possible use in preventing thermal injury during RF ablation [13–16]. These devices provided flow rates of 25 mL to 300 mL per minute, and although preclinical as well as mathematical models of the devices suggested benefits in lesion reduction, none of the early prototypes evolved into commercially available products. A more recently developed intraesophageal cooling device has a flow rate of up to 1900 mL per minute. This device was designed for whole-body temperature manipulation, for which it is commercially available, and has been shown to have protective effects in animal and mathematical models of RF ablation. It is currently under clinical investigation for use during RF ablation, and preclinical data suggest a close correlation between water temperature and protective effect, such that increased heat extraction results in greater reduction in lesion thickness [22, 23]. Recent data in the burn literature also suggests that there are improved outcomes from thermal burns (reduced full thickness depth, skin grafting requirement, hospitalization, and other operative interventions) with cooling, with a dose-response relationship noted between the odds of grafting and duration of cool running water, which may offer further support for the idea of a threshold effect to preventing progression of thermal injury after the initial thermal insult [24].

A recent meta-analysis of two studies that used the direct instillation of ice-cold water to cool the esophagus focused on the outcome of overall lesion frequency, but did not distinguish between lesion severity and lesion frequency, and produced inconclusive results [25]. In contrast, in our meta-analysis of 3 studies that used this cooling method, we stratified the lesions according to their severity and found that cooling by this method reduced the number of high-grade lesions. Although the mechanism of AEF formation is not well understood, there is general agreement that thermal injury is a precursor and that higher-grade thermal injury has a higher risk of progression to AEF [26].

Analyzing each lesion grade independently in a statistical model allows an alternative approach to estimate effect size that may provide further refinement of the estimate, at the cost of decreased precision of the estimate. The point estimate that we found using this approach suggests a reduction in high-grade lesions of −23%, although a higher number of patients with a greater number of high-grade lesions would be necessary to narrow the confidence intervals around this estimate, which, with the population included here, ranges from −85% to +38%. It seems likely that cooling methods that have a higher heat extraction capacity will lead to increased effect size point estimates when the data are analyzed by either binary logistic or ordinal regression.

## Limitations

The three studies that we analyzed differed in patient characteristics and other details as well as in the specific radiofrequency techniques and equipment used (see Tables 1, 2, 3). Nevertheless, all studies used radiofrequency ablation, and the variation in technology reflects current real-world practice. One of the studies randomized the patients. There was no description of any attempt at blinding the patients to the protection strategy used. Lesion grading varied between studies, but the scales used in each study fitted easily into a common stratification. The differences between the studies may serve to broaden the generalizability of this meta-analysis.

## Conclusions

Esophageal cooling reduces the severity of the lesions that may result from RF ablation, even when relatively low heat extraction methods are used, such as the direct instillation of small amounts of cold liquid. Further investigation of esophageal cooling is warranted, particularly with higher heat extraction capacity techniques.

## References

[1] Romero J, Avendano R, Grushko M, Diaz JC, Du X, Gianni C, Natale A, Biase LD (2018). Oesophageal injury during AF ablation: techniques for prevention. Arrhythm Electrophysiol Rev.

[2] Kapur S, Barbhaiya C, Deneke T, Michaud GF (2017). Esophageal injury and atrioesophageal fistula caused by ablation for atrial fibrillation. Circulation.

[3] Qumseya BJ, Kusumoto F, Wolfsen H (2012). Esophageal injury following left atrial ablation. Gastroenterol Hepatol (N Y).

[4] Khakpour H, Shemin RJ, Lee JM, Buch E, Boyle NG, Shivkumar K, Bradfield JS (2017). Atrioesophageal fistula after atrial fibrillation ablation: a single center series. Journal of atrial fibrillation.

[5] Yousuf T, Keshmiri H, Bulwa Z, Kramer J, Sharjeel Arshad HM, Issa R, Woznicka D, Gordon P, Abi-Mansour P (2016). Management of atrio-esophageal fistula following left atrial ablation. Cardiol Res.

[6] Khan MY, Siddiqui WJ, Iyer PS, Dirweesh A, Karabulut N (2016). Left atrial to esophageal fistula: a case report and literature review. The American journal of case reports.

[7] Nair GM, Nery PB, Redpath CJ, Lam BK, Birnie DH (2014). Atrioesophageal fistula in the era of atrial fibrillation ablation: a review. Can J Cardiol.

[8] Zakaria A, Hipp K, Battista N, Tommolino E, Machado C: Fatal esophageal-pericardial fistula as a complication of radiofrequency catheter ablation. SAGE open medical case reports 2019, 7:2050313x19841150. 10.1177/2050313X19841150 PMC6452424 31057797

[9] Deviating the esophagus in atrial fibrillation ablation - clinicaltrials.gov listing [https://clinicalt rials.gov/ct2/show/NCT01546168]. Accessed 13 Sep 2019

[10] Kadado AJ, Akar JG, Hummel JP (2019). Luminal esophageal temperature monitoring to reduce esophageal thermal injury during catheter ablation for atrial fibrillation: a review. Trends in Cardiovascular Medicine.

[11] Deneke T, Bunz K, Bastian A, Pasler M, Anders H, Lehmann R, Meuser W, de Groot JR, Horlitz M, Haberkorn R (2011). Utility of esophageal temperature monitoring during pulmonary vein isolation for atrial fibrillation using duty-cycled phased radiofrequency ablation. J Cardiovasc Electrophysiol.

[12] Berjano EJ, Hornero F (2005). A cooled intraesophageal balloon to prevent thermal injury during endocardial surgical radiofrequency ablation of the left atrium: a finite element study. Phys Med Biol.

[13] Lequerica JL, Berjano EJ, Herrero M, Hornero F (2008). Reliability assessment of a cooled intraesophageal balloon to prevent thermal injury during RF cardiac ablation: an agar phantom study. J Cardiovasc Electrophysiol.

[14] Lequerica JL, Berjano EJ, Herrero M, Melecio L, Hornero F (2008). A cooled water-irrigated intraesophageal balloon to prevent thermal injury during cardiac ablation: experimental study based on an agar phantom. Phys Med Biol.

[15] Arruda MS, Armaganijan L, Di Biase L, Rashidi R, Natale A (2009). Feasibility and safety of using an esophageal protective system to eliminate esophageal thermal injury: implications on atrial-esophageal fistula following AF ablation. J Cardiovasc Electrophysiol.

[16] Tsuchiya T, Ashikaga K, Nakagawa S, Hayashida K, Kugimiya H (2007). Atrial fibrillation ablation with esophageal cooling with a cooled water-irrigated intraesophageal balloon: a pilot study. J Cardiovasc Electrophysiol.

[17] Scanavacca MI, Pisani CF, Neto S, Tamaki W, Santo SR, Guirao C, Oyama H, Aielo V, Leiner A, Sosa E (2007). Cooled intra-esophageal balloon to prevent thermal injury of esophageal wall during radiofrequency ablation. In: ESC Congress 2007, 1 - 5 September. vol. 28. Vienna, Austria.

[18] Kuwahara T, Takahashi A, Okubo K, Takagi K, Yamao K, Nakashima E, Kawaguchi N, Takigawa M, Watari Y, Sugiyama T (2014). Oesophageal cooling with ice water does not reduce the incidence of oesophageal lesions complicating catheter ablation of atrial fibrillation: randomized controlled study. Europace.

[19] Sohara H, Satake S, Takeda H, Yamaguchi Y, Nagasu N (2014). Prevalence of esophageal ulceration after atrial fibrillation ablation with the hot balloon ablation catheter: what is the value of esophageal cooling?. J Cardiovasc Electrophysiol.

[20] John J, Garg L, Orosey M, Desai T, Haines DE, Wong WS. The effect of esophageal cooling on esophageal injury during radiofrequency catheter ablation of atrial fibrillation. J Interv Card Electrophysiol. 2019. 10.1007/s10840-019-00566-3 31154536

[21] Luchini C, Stubbs B, Solmi M, Veronese N (2017). Assessing the quality of studies in meta-analyses: Advantages and limitations of the Newcastle Ottawa Scale. World J Meta-Anal.

[22] Montoya MM, Mickelsen S, Clark B, Arnold M, Hanks J, Sauter E, Kulstad E (2019). Protecting the esophagus from thermal injury during radiofrequency ablation with an esophageal cooling device. Journal of atrial fibrillation.

[23] Feher M, Anneken L, Gruber M, Achenbach S, Arnold M (2018). Esophageal cooling for prevention of thermal lesions during left atrial ablation procedures: a first in man case series. In: EHRA 2019: March 19, 2019 2018; Lisbon, Portugal.

[24] Griffin BR, Frear CC, Babl F, Oakley E, Kimble RM. Cool running water first aid decreases skin grafting requirements in pediatric burns: a cohort study of two thousand four hundred ninety-five children. Ann Emerg Med. 2019. 10.1016/j.annemergmed.2019.06.028 31474480

[25] Ha FJ, Han HC, Sanders P, Teh AW, O'Donnell D, Farouque O, Lim HS (2019). Prevalence and prevention of oesophageal injury during atrial fibrillation ablation: a systematic review and meta-analysis. Europace.

[26] Halbfass P, Pavlov B, Muller P, Nentwich K, Sonne K, Barth S, et al. Progression from esophageal thermal asymptomatic lesion to perforation complicating atrial fibrillation ablation: a single-center registry. Circ Arrhythm Electrophysiol. 2017;10(8). 10.1161/CIRCEP.117.005233 28798021

